# From Large-Scale Characterization to Subgroup-Specific Predictive Modeling: A Study on the Diagnostic Value of Liver Stiffness Measurements in Focal Liver Lesions

**DOI:** 10.3390/diagnostics15161986

**Published:** 2025-08-08

**Authors:** Ying Xu, Ying-Long Guo, Qian-Yu Lv, Zheng Wang, Jian Zhou, Jie Hu

**Affiliations:** 1 School of Health Science and Engineering, University of Shanghai for Science and Technology, Shanghai 200093, China; 2 Liver Cancer Institute, Zhongshan Hospital, Fudan University, Shanghai 200032, China; 3 Key Laboratory of Carcinogenesis and Cancer Invasion, Ministry of Education, Shanghai 200032, China; 4 Department of Radiology, Zhongshan Hospital, Fudan University, Shanghai 200032, China

**Keywords:** liver stiffness measurements, liver lesions, stiffness features

## Abstract

**Background/Objectives:** As a noninvasive indicator of liver fibrosis and stiffness, liver stiffness measurement (LSM) has also shown significant value in differentiating focal liver lesions (FLLs). This study aimed to assess the characteristics of LSM values across different liver lesions and explore their value in differential diagnosis. **Methods:** A total of 8817 individuals with FLLs were assessed using liver stiffness measurements (LSMs). We evaluated the LSM characteristics across different FLL categories and further compared these values within subgroups based on their alpha-fetoprotein (AFP) and hepatitis B surface antigen (HBsAg). The LSM was visualized graphically. We compared two logistic regression models (with the LSM and without the LSM) in a cohort of 2271 patients who were both AFP-normal (<20 ng/mL) and HBsAg-negative. The differentiation value of the LSM was quantified by comparing the models’ area under the curves (AUCs) and through decision curve analysis (DCA). **Results:** The LSM showed significant differences (*p* < 0.001) among malignant lesions, benign lesions, and cirrhotic nodules (CN). Among benign lesions, only focal nodular hyperplasia (FNH) and simple hepatic cysts (SHC) showed a significant difference (*p* < 0.05). Among malignant lesions, significant differences in the LSM were observed between all pairs (*p* < 0.001) except between hepatocellular carcinoma (HCC) and combined hepatocellular-cholangiocarcinoma (cHCC-CC). Patients with elevated AFP levels exhibited significantly higher LSM across most lesion types. HBsAg-positive patients also showed significantly increased LSM in all five lesion types, except for CN and cHCC-CC. The full model (with LSM) for differentiating primary malignant lesions from benign ones was built using six variables. The AUCs of the full model were 0.897 and 0.896 in the training and validation sets, significantly outperforming the comparison model (AUC: 0.882, *p* = 0.0002; 0.879, *p* = 0.017). **Conclusions:** The LSM can provide additional information on focal liver lesions.

## 1. Introduction

Liver stiffness measurement (LSM) is a noninvasive method for assessing liver fibrosis and cirrhosis, and is widely used in clinical practice [1,2,3]. In recent years, LSM has also been investigated for its value in evaluating focal liver lesions and has been shown to reflect tissue stiffness [4,5,6].

Focal liver lesions (FLLs) are abnormal solid or cystic masses or tissue areas within the liver, ranging from benign tumors to malignant diseases such as primary liver cancer (PLC) [7]. FLLs are frequently detected during routine examinations. PLC is a major cause of cancer-related deaths and has a relatively high incidence in China [8,9]. Serum alpha-fetoprotein (AFP) is commonly used for the screening of PLC [10,11]. Elevated AFP levels often indicate the presence of hepatocellular carcinoma (HCC). Hepatitis B surface antigen (HBsAg) reflects hepatitis B virus (HBV) infection, which is a major risk factor for HCC. HBV has a high prevalence in China [12]. Without treatment, chronic HBV infection may lead to fibrosis and eventually cirrhosis. Although liver biopsy is regarded as the gold standard for diagnosing FLLs, it carries risks of bleeding and an increased probability of severe complications and mortality [13]. Therefore, employing less invasive techniques such as LSM to characterize these lesions can provide valuable supplementary diagnostic information.

In the current study, we aimed to analyze the characteristics and distribution of LSM in different types of FLLs. LSM between subgroups stratified by serum alpha-fetoprotein (AFP) and hepatitis B surface antigen (HBsAg) status was also compared. Additionally, we aimed to evaluate the diagnostic value of LSM for differentiating primary malignant lesions within the subgroup of patients negative for both AFP and HBsAg.

## 2. Materials and Methods

**Figure 1:**
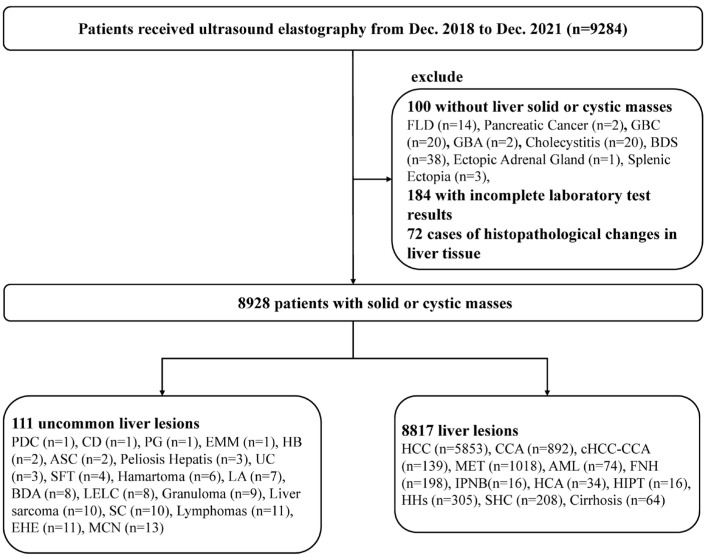
Flow chart of patient inclusion and exclusion. FLD, fatty liver disease; GBC, gallbladder cancer; GBA, gallbladder adenoma; BDS, bile duct stones; PDC, poorly differentiated carcinoma; CD, Caroli’s disease; PG, paraganglioma; EMM, epithelioid malignant mesothelioma; HB, hepatoblastoma; ASC, adenosquamous carcinoma; UC, undifferentiated carcinoma; SFT, solitary fibrous tumor; LA, liver abscess; BDA, bile duct adenoma; LELC, lymphoepithelioma-like carcinoma; SC, sarcomatoid carcinoma; EHE, epithelioid hemangioendothelioma; MCN, mucinous cystic neoplasms; HCC, hepatocellular carcinoma; CCA, cholangiocarcinoma; cHCC-CCA, combined heapatocellular-cholangiocarcinoma; MET, metastatic carcinoma; AML, hepatic angiomyolipoma; FNH, focal nodular hyperplasia; IPNB, intraductal papillary neoplasms of bile duct; HCA, hepatocellular adenoma; HIPT, hepatic inflammatory pseudotumor; HHs, hepatic hemangiomas; SHC, simple hepatic cysts; CN, cirrhotic nodules.

A total of 9284 hospitalized patients who underwent elastography at Zhongshan Hospital, Fudan University, from December 2018 to December 2021, were retrospectively collected. After excluding 172 individuals without liver solid or cystic lesions and 184 individuals with incomplete laboratory test results, 8928 patients were enrolled in the study. Considering the low incidence of some liver lesions in the clinical setting, the limited sample size, and the complexity of histological sources, the 111 uncommon liver lesions in this study were grouped separately. These lesions were analyzed descriptively and excluded from the statistical analysis. Finally, the study mainly focused on 8817 patients with common liver lesions (Figure 1).

For the 8817 patients with FLLs, we collected their clinical data, serological results, and liver stiffness measurements (LSMs). LSM values were obtained using 2D shear wave elastography (2D-SWE) performed on an Aixplorer ultrasound imaging system (Supersonic Imagine, Aix-en-Provence, France), equipped with a SC6–1 convex probe. Following a standardized protocol, five valid measurements were acquired for each patient, and the mean value was calculated and recorded as the final LSM. All lesions were confirmed by pathological diagnosis or imaging diagnosis.

This study has been approved by our ethical committee, and as patient information was anonymous, informed consent was not required. This study was conducted in accordance with the Declaration of Helsinki.

### 2.1. Features of Liver Stiffness Measurements in Liver Lesions

Liver lesions in the study cohort were described and compared according to different criteria: 1. We divided all patients into three categories according to the nature of the lesions: benign lesions, malignant lesions, and cirrhotic nodules, and compared the differences in LSM between the three categories; 2. In the group of benign and malignant lesions, the LSM values were compared across different lesion types; 3. According to clinical thresholds of serological indicators, each type of liver lesion was divided into normal and abnormal groups to compare their LSM values.

### 2.2. Diagnostic Value of Liver Stiffness Measurements in AFP and HBsAg-Negative Cohort

A key focus of this study was to evaluate a diagnostically challenging cohort. Our initial descriptive analysis revealed that some primary malignant lesions presented with serum markers within normal ranges. This made it difficult to differentiate them from benign lesions. Therefore, we conducted a subgroup analysis focused on patients who were serologically normal/negative. Specifically, it included individuals with a normal serum Alpha-fetoprotein (AFP) level, defined as <20 ng/mL, and a negative Hepatitis B surface antigen (HBsAg) status, defined as <0.05 IU/mL, based on standard clinical thresholds.

This subgroup analysis subsequently included all patients who met the following criteria: (1) a pathologically confirmed primary liver lesion (metastatic tumors excluded); (2) a normal AFP status; and (3) a negative HBsAg status. A total of 2271 patients who fulfilled these conditions were included in this analysis. A diagnostic model combining LSM and serological markers was constructed to distinguish primary benign from malignant lesions. The cohort was randomly divided into training and validation sets at a ratio of 8:2.

We constructed and compared two multivariable logistic regression models. The full model included LSM, clinical data, serological indicators. To enhance the model’s clinical utility and interpretability, certain continuous variables were dichotomized based on established, widely recognized clinical reference ranges (values were considered abnormal if they met the following thresholds, which are based on common clinical reference ranges: aspartate aminotransferase (AST) > 40 U/L; γ-glutamyl transferase (GGT) > 50 U/L; alanine aminotransferase (ALT) > 50 U/L; total bilirubin (TBIL) > 17.1 μmol/L; des-gamma-carboxy prothrombin (DCP) > 20 μg/L; prothrombin time (PT) > 13 s; international normalized ratio (INR) > 1.0; platelet count (PLT) < 100 × 10^9^/L).

A comparison model was developed by excluding the LSM. In the training set, candidate variables were screened using the least absolute shrinkage and selection operator (LASSO) method combined with ten-fold cross-validation. Based on the selected variables, both models were constructed with multivariable logistic regression. Model performance was assessed by the area under the curve (AUC), the Hosmer–Lemeshow goodness-of-fit test, and decision curve analysis (DCA). The AUCs of the two models were statistically compared using DeLong’s test.

### 2.3. Statistical Analysis

Statistical analyses were performed by R software (version 4.2.3), SPSS software (version 15.0), and GraphPad Prism (version 9.0). Continuous variables were expressed as medians (the interquartile range, IQR) or means ± SD and compared by the t-test or Mann–Whitney U test. Categorical variables were expressed as the number (percentage) and were compared using the chi-squared test. A *p* < 0.05 was considered statistically significant.

## 3. Results

### 3.1. Liver Stiffness Measurements in 8817 Liver Lesions

**Figure 2:**
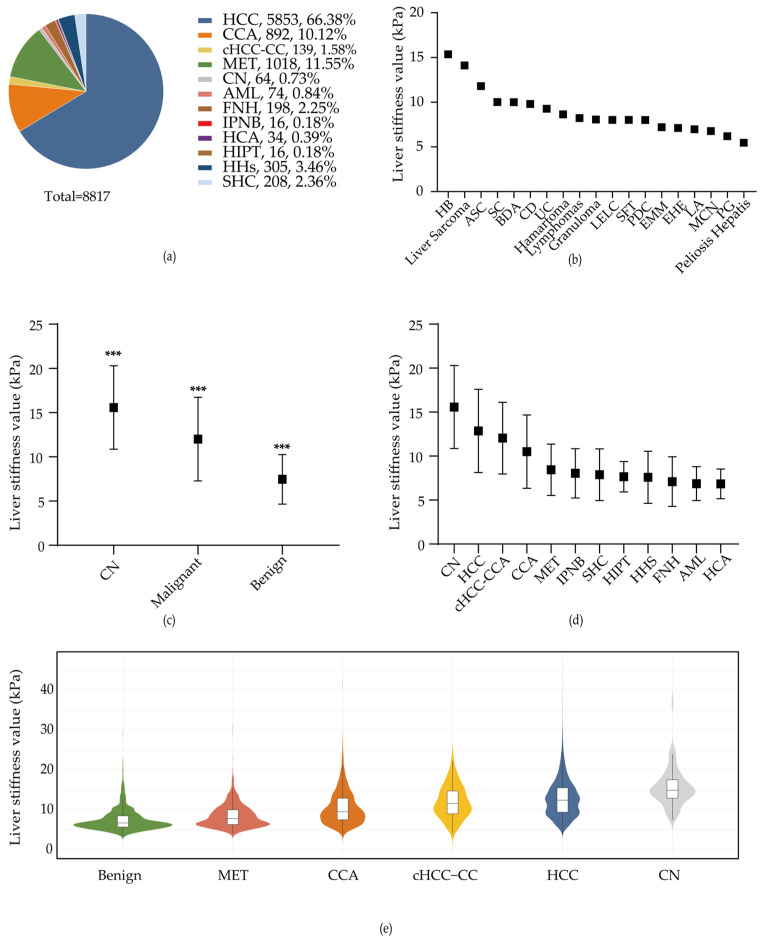
(**a**) Distribution of all types of FLLs among the 8817 patients; (**b**) liver stiffness measurement (LSM) of 111 uncommon liver lesions; (**c**) comparison of mean LSM values among benign lesions, malignant lesions, and CN according to the mean of liver stiffness measurements, *** indicates significant differences between each pair of groups; (**d**) comparison of all kinds of lesions in 8817 liver lesions according to the mean of LSM values; (**e**) the distribution of LSM in different FLLs, a case of HCC with an LSM value of 88.6 kPa was excluded from the plot for visualization purposes.

The liver stiffness measurements of the three subgroups (benign lesions, malignant lesions, and cirrhotic nodules) are summarized in Table 1 and Figure 2c. The analysis revealed that cirrhotic nodules had significantly higher stiffness values than both malignant and benign liver lesions. Consequently, cirrhotic nodules were classified as a separate group due to their stiffness. The LSM values increased progressively from benign to malignant lesions, with cirrhotic nodules showing the highest values. A statistically significant difference in LSM was observed among benign lesions, malignant lesions, and cirrhotic nodules (*p* < 0.001).

#### 3.1.1. Liver Stiffness Measurements in Various Liver Lesion Types in Benign and Malignant Lesions

The stiffness values of all types of liver lesions in 8817 patients are summarized in Table 2 and Figure 2d. Among the benign lesions, intraductal papillary neoplasms (IPNB) had the highest mean LSM, followed by simple hepatic cysts (SHC). A significant difference was observed only between focal nodular hyperplasia (FNH) and SHC (*p* < 0.05). Hepatocellular carcinoma (HCC) showed the highest stiffness among all malignant lesions, followed by combined hepatocellular-cholangiocarcinoma (cHCC-CC). Metastatic carcinoma (MET) showed the lowest LSM. Significant differences in the LSM were observed between each pair of the four malignant lesions (*p* < 0.001), except for the comparison between HCC and cHCC-CC, which showed no significant difference (*p* > 0.05).

Figure 2e illustrates that benign lesions exhibit extremely high data density below the normal threshold (<7.5 kPa). In contrast, most malignant lesions and cirrhotic nodules, except metastatic carcinoma (MET), are sparsely distributed within this range.

#### 3.1.2. Liver Stiffness Measurements of 8817 Liver Lesions Under Subgroups of Serology

**Figure 3:**
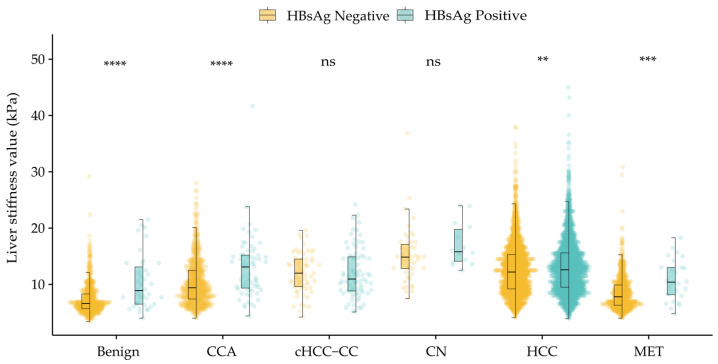
Distribution of liver stiffness measurements in each type of focal liver lesion in the subgroups of hepatitis B surface antigen (HBsAg). Each dot represents an individual patient’s LSM value. The overlaid black boxplot illustrates the median and interquartile range (IQR), and extends whiskers to 1.5 times the IQR. Statistical significance is denoted as: ns, *p* > 0.05; **, *p* ≤ 0.01; ***, *p* ≤ 0.001; ****, *p* ≤ 0.0001. One case of HCC with an LSM value of 88.6 kPa was excluded from the plot.

- Subgroups based on HBsAg (Figure 3)

Among CN, cHCC-CC, and HCC, the proportion of HBsAg-positive cases was higher, whereas the HBsAg-negative cases accounted for a greater share in benign lesions, MET, and CCA. Across all lesion types, the HBsAg-positive groups showed higher LSM values. Except for CN and cHCC-CC (*p* > 0.05), there were significant differences between the HBsAg-positive and HBsAg-negative groups in the remaining four lesion types.

**Figure 4:**
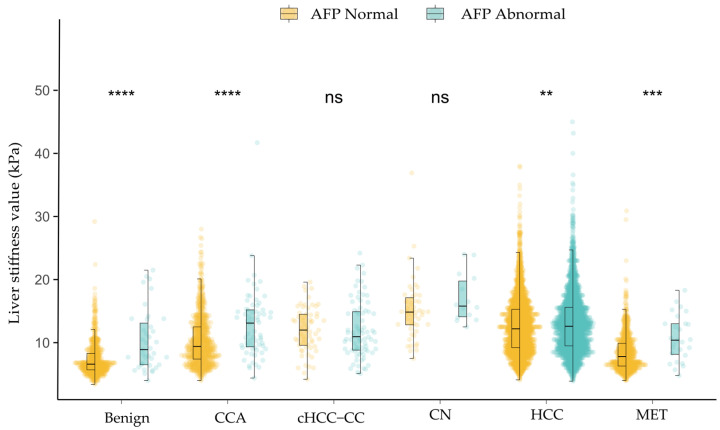
Distribution of liver stiffness measurement in each type of focal liver lesion in the subgroups of alpha-fetoprotein (AFP). Each dot represents an individual patient’s LSM value. The overlaid black boxplot illustrates the median and interquartile range (IQR), and extends whiskers to 1.5 times the IQR. Statistical significance is denoted as: ns, *p* > 0.05; **, *p* ≤ 0.01; ***, *p* ≤ 0.001; ****, *p* ≤ 0.0001. One case of HCC with an LSM value of 88.6 kPa was excluded from the plot.

- Subgroups based on AFP (Figure 4)

According to the clinical threshold of 20 ng/mL, patients were classified as normal (<20 ng/mL) or elevated (≥20 ng/mL). Across all six lesion categories, the proportion of cases with normal AFP levels (<20 ng/mL) was higher than those with elevated AFP. Lesions in the normal AFP group consistently showed lower LSM values compared to those with abnormal AFP. Significant differences in LSM between AFP groups were observed in benign lesions, HCC, CCA, and MET (*p* < 0.05), while no significant differences were found in CN and cHCC-CC (*p* > 0.05).

### 3.2. Diagnostic Value of Liver Stiffness Measurements in AFP and HBsAg Negative Cohort

A total of 2271 patients were included in this study and randomly divided into a training set (*n* = 1818) and a validation set (*n* = 453). As shown in Table 3, there were no significant differences between the training and validation sets in terms of age, gender, serum parameters, or LSM (*p* > 0.05).

The results of the correlation analysis between the LSM and serological markers are provided in the Supplementary Materials (Supplementary Table S1 and Supplementary Figure S1).

**Figure 5:**
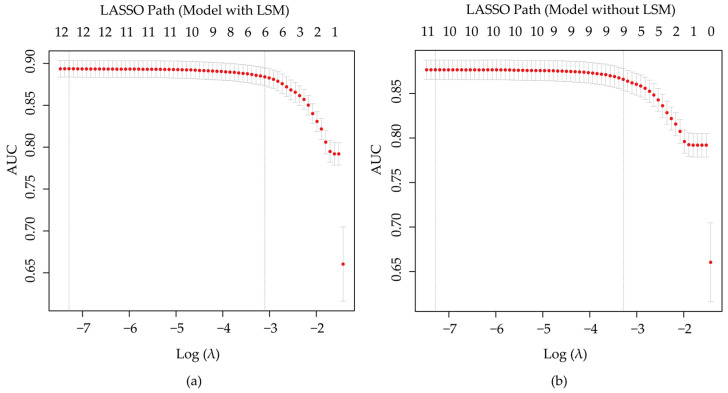
Least absolute shrinkage and selection operator (LASSO) regression combined with ten-fold cross-validation for feature selection in the full model (**a**) and the comparison model (**b**). In both plots, the *x*-axis represents the logarithm of the penalty parameter (λ), and the *y*-axis shows the average area under the receiver operating characteristic (ROC) curve (AUC) derived from ten-fold cross-validation. The numbers along the top axis indicate the number of variables retained in the model at each λ value. The optimal λ was selected using the “1 standard error” rule (lambda.1se), which balances model simplicity and performance, and was used to determine the final set of predictors.

In the training set, LASSO regression with ten-fold cross-validation was used to select variables for both the full model (with LSM) and the comparison model (without LSM) (Figure 5). For the full model, six variables were identified: LSM, age, gender, AST, GGT, and DCP. Eight variables (age, gender, AST, GGT, DCP, PLT, PT, and INR) were selected for the comparison model. Finally, these selected variables were included in multivariate logistic regression to construct the diagnostic models for differentiating primary malignant lesions.

The multivariate logistic regression analysis results are presented in Table 4. In the full model, LSM, age, gender, AST, GGT, and DCP were independently associated with malignant lesions. In the comparison model, PLT, PT, and INR were also identified as independent predictors.

**Figure 6:**
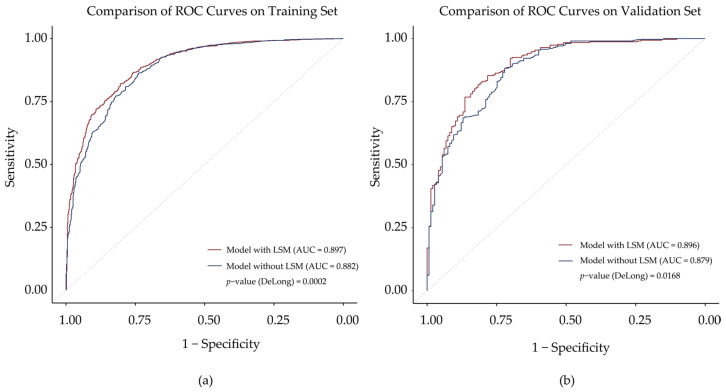
Receiver operating characteristic (ROC) curves for the full model (with LSM) and comparison model (without LSM) in the training set (**a**) and validation set (**b**). The calibration curves demonstrated good agreement between the predicted and observed probabilities for both the full and comparison models in the validation cohort (Figure 7). The Hosmer–Lemeshow test also supported the adequate model fit (full model: *p* = 0.517; the comparison model: *p* = 0.440).

To evaluate the models in differentiating primary malignant lesions, receiver operating characteristic (ROC) curve analysis was utilized for both the training and validation sets. The full model (with LSM) achieved AUCs of 0.897 and 0.896 in the training and validation sets, respectively. The comparison model (without LSM) had AUCs of 0.882 and 0.879. In both training and validation sets, DeLong’s test confirmed that the full model outperformed the comparison model with a statistically significant difference (AUC: 0.897 vs. 0.882, *p* = 0.0002; 0.896 vs. 0.879, *p* = 0.017) (Figure 6).

**Figure 7:**
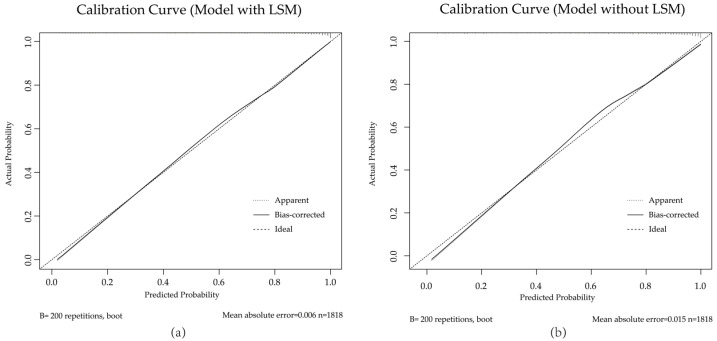
Calibration curves of the full model (with LSM) (**a**) and comparison model (without LSM) (**b**) in the validation set. Both models demonstrate good agreement between predicted and observed probabilities. The dashed line indicates perfect calibration.

**Figure 8:**
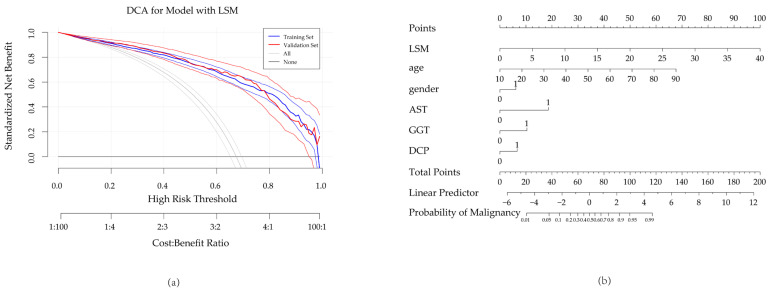
(**a**) Decision curve analysis showed that the full model provided a net clinical benefit across a wide range of threshold probabilities (0.1–0.9), confirming its practical utility. The bold blue line shows the model’s net benefit for the training set. The bold red line shows it for the validation set. Their thin lines indicate the 95% confidence intervals. The grey lines represent the net benefit if all lesions are assumed malignant. This includes their 95% confidence interval. The black horizontal line signifies the net benefit if no lesions are assumed malignant.; (**b**) a nomogram based on the full model, the total points, derived by summing the points for each predictor, correspond to a specific predicted risk on the bottom axis. Predictors include LSM (kPa), AST (0: ≤40 U/L, 1: >40 U/L), GGT (0: ≤50 U/L, 1: >50 U/L), and DCP (0: ≤20 μg/L, 1: >20 μg/L).

Decision curve analysis (Figure 8a) showed that the full model provided a net clinical benefit across a wide range of threshold probabilities (0.1–0.9), confirming its practical utility. Based on the full model, we constructed a nomogram for clinical prediction (Figure 8b).

## 4. Discussion

As a quantitative biomarker reflecting changes in the liver’s biomechanical properties, liver stiffness measurement (LSM) is widely used in the clinical assessment of liver fibrosis [1]. Based on this, our study aimed to further investigate the state of different focal liver lesions using LSM. The results showed that the LSM values were significantly different among the three groups of benign lesions, malignant lesions, and cirrhotic nodules. Cirrhotic nodules were categorized separately in this study to avoid interference in differentiating benign and malignant lesions. In the comparison of benign and malignant lesions, the LSM values were significantly higher in malignant lesions. Comparable findings have been reported in previous studies on liver stiffness in focal liver lesions [5,14,15]. The increased liver stiffness in malignant lesions may be attributed to their dense cellular architecture and invasive growth patterns. At the microstructural level, factors such as elevated cellular density, altered nucleocytoplasmic ratio, and actin cytoskeleton remodeling further contribute to a more rigid tumor microenvironment [14,16,17,18]. The highest LSM values were observed in HCC, consistent with the findings of Keskin et al. [19]. However, some studies [20,21] reported the highest stiffness in cholangiocarcinoma (CCA). This discrepancy may be related to differences in the liver conditions. HCC often develops in the context of severe fibrosis or cirrhosis, which significantly affects the LSM [22,23,24]. Another important reason may be the difference in the sample size of the studies. Most of the metastatic carcinomas (MET) cases still exhibited normal stiffness values (<7.5 kPa). This can be largely attributed to the fact that METs typically arise in non-cirrhotic or non-fibrotic liver parenchyma. Additionally, the intrinsic stiffness of these lesions varies considerably, depending on the histology of the primary tumor. Among the benign lesions, IPNB exhibited the highest LSM. This may be related to its characteristics as a benign, preinvasive, intraductal tumor that originates from the biliary epithelium but is considered a precursor to malignant growth [25,26].

In this study, we focused on the effects of the HBsAg and AFP statuses on the LSM. HBsAg reflects the disease status of chronic hepatitis B (CHB) infections and may contribute to the development of cirrhosis and HCC and increase liver stiffness [27]. Liver inflammation or injury activates hepatic stellate cells (HSCs) to produce and accumulate extracellular matrix (ECM), promoting fibrosis [28,29]. There were significant differences in LSM between the HBsAg-positive and HBsAg-negative groups in benign lesions, HCC, CCA, and MET. However, serum AFP levels remain normal in 15–30% of advanced HCC cases, and some benign diseases can also lead to elevated AFP levels [30,31]. This may be because the AFP levels correlate with the fibrosis stages, with higher levels of liver fibrosis often accompanied by elevated AFP levels [32,33]. AFP levels increase not only in hepatocellular carcinoma but also in response to inflammation and fibrosis [34,35]. This may explain why the LSM values were higher in the normal AFP group compared to the abnormal group in benign lesions and MET.

We observed that in the subgroup of AFP-normal or HBsAg-negative, some primary malignant lesions still exhibited high LSM values. A previous study has noticed AFP-negative hepatic lesions and developed effective diagnostic models based on commonly available serologic markers [36]. However, the model included HBV infection as a predictor, limiting its applicability to HBsAg-negative populations. In our study, we focused on primary liver lesions that were both AFP-normal and HBsAg-negative.

The diagnostic value of liver stiffness for distinguishing benign and malignant lesions has been widely demonstrated [5,21,37]. However, few studies have evaluated its ability to differentiate benign from malignant lesions in patients negative for both AFP and HBsAg. This population represents a common and significant diagnostic challenge in clinical practice. In these individuals, conventional serological markers are often non-contributory, a limitation that can lead to diagnostic delays. The full model achieved an AUC of 0.896 for distinguishing primary malignant from benign lesions, significantly outperforming the comparison model without the LSM (AUC = 0.879, *p* = 0.017). These results confirm the clinical relevance of the LSM in patients both with AFP-normal and HBsAg-negative. Our full model (with LSM) showed good discrimination ability, model fit, and strong clinical utility. AST, GGT, DCP, gender, and age were included as independent predictors in the model. They were well-recognized risk factors or markers associated with hepatocellular injury and the development of liver cancer [38,39,40,41].

These indicators in the models were selected by LASSO regression [42,43]. Although some information may be lost by dichotomizing continuous serological variables, this approach enhances the interpretability of clinical results. It also greatly facilitates the subsequent construction of the visualized nomogram tool. As a retrospective study conducted at a single center, the results may be influenced by selection bias. The general ability of the model requires further validation using external datasets from multiple centers.

## 5. Conclusions

We investigated the stiffness characteristics and fibrosis distribution in liver lesions using the LSM. The LSM provides additional insights into focal liver lesions and, when combined with serum markers, enhances the accuracy in differentiating primary malignant lesions from benign lesions.

## Figures and Tables

**Table 1:** Liver stiffness measurement of 8817 liver lesions.

	Number (%)	LSM (kPa)	
		Median (Min.–Max.)	Mean ± SD
Cirrhotic nodules *	64 (0.73)	14.90 (7.50–36.90)	15.58 ± 4.72
Malignant *	7902 (89.62)	11.39 (3.90–88.60)	12.02 ± 4.73
Benign *	851 (9.65)	6.67 (3.40–29.20)	7.46 ± 2.80

* indicates significant differences between each pair of groups; LSM, liver stiffness measurements.

**Table 2:** Liver stiffness measurements of various liver lesion types.

	Number (%)	LSM (kPa)	
		Median (Min.–Max.)	Mean ± SD
Malignant	7902		
HCC *^#^	5853 (74.0)	12.40 (3.90–88.60)	12.87 ± 4.73
CCA *	892 (11.29)	9.53 (4.00–41.70)	10.51 ± 4.17
cHCC-CC *^#^	139 (1.76)	11.55 (4.20–24.20)	12.05 ± 4.07
MET *	1018 (12.88)	7.83 (4.00–30.90)	8.44 ± 2.91
Benign	851		
AML	74 (8.70)	6.53 (3.40–12.50)	6.87 ± 1.92
FNH **	198 (23.27)	6.20 (3.60–21.50)	7.10 ± 2.83
IPNB	16 (1.88)	7.27 (4.40–13.10)	8.05 ± 2.80
HCA	34 (4.00)	6.30 (4.60–10.80)	6.84 ± 1.68
HIPT	16 (1.88)	7.85 (5.10–11.40)	7.66 ± 1.72
HHs	305 (35.84)	6.66 (3.60–22.40)	7.59 ± 2.96
SHC **	208 (24.44)	6.96 (4.10–29.20)	7.88 ± 2.94
Cirrhotic nodules	64 (0.73)	14.90 (7.50–36.90)	15.58 ± 4.72

LSM, liver stiffness measurement; *, a significant difference between the two groups in malignant lesions exists; ^#^, no significant difference between the HCC and cHCC-CCA; **, there exists a significant difference between the FNH and SHC.

**Table 3:** Comparison of clinical information, laboratory parameters, and liver stiffness values between the training set and validation set.

	Training (*n* = 1818)	Validation (*n* = 453)	*p* Value
Age	59.69 (14.04)	59.85 (14.78)	0.883
Gender (%)			0.561
Male	1157 (63.6%)	281 (62.0%)	
Female	661 (36.4%)	172 (38.0%)	
LSM (kPa)	9.88 (4.44)	9.75 (4.26)	0.577
DCP (μg/L)	844.07 (4704.99)	726.92 (4761.34)	0.636
PLT (×10^9^/L)	195.93 (79.33)	200.59 (80.45)	0.265
TBIL (μmol/L)	21.40 (41.53)	24.09 (52.67)	0.244
ALT (U/L)	36.08 (56.84)	35.89 (57.08)	0.947
AST(U/L)	34.30 (48.95)	32.08 (34.17)	0.364
GGT (U/L)	118.88 (233.07)	138.66 (281.62)	0.122
PT (s)	11.88 (1.23)	11.88 (1.14)	0.959
INR	1.05 (0.11)	1.05 (0.10)	0.827

LSM, liver stiffness measurements; DCP, des-gamma-carboxy prothrombin; PLT, platelet count;; TBIL, total bilirubin; AST, aspartate aminotransferase; ALT, alanine aminotransferase; GGT, γ-glutamyl transferase; PT, prothrombin time; INR, international normalized ratio.

**Table 4:** Multivariable logistic regression analysis of risk factors for primary malignant lesions.

	Full Model (LSM) OR (95% CI)	Comparison Model (No LSM) OR (95% CI)
LSM	1.27 (0.03) ***	
age	1.08 (0.01) ***	1.10 (0.01) ***
gender	1.79 (0.14) ***	2.06 (0.14) ***
AST > 40 U/L	5.87 (0.33) ***	7.03 (0.31) ***
GGT > 50 U/L	2.68 (0.15) ***	2.95 (0.15) ***
DCP > 20 μg/L	1.89 (0.15) ***	2.35 (0.15) ***
PLT < 100 × 10^9^/L		3.12 (0.33) ***
PT > 13 s		2.51 (0.32) **
INR > 1		1.57 (0.14) **

*** *p* < 0.001, ** *p* < 0.01.

## Data Availability

The data presented in this study are not publicly available but are available upon request from the corresponding author. The data are not publicly available due to privacy and ethical restrictions.

## Supplementary Materials

The following supporting information can be downloaded at: https://www.mdpi.com/article/10.3390/diagnostics15161986/s1, Table S1: Spearman’s correlation analysis of liver stiffness measurement (LSM) with serological indicators; Figure S1: Correlation analysis between liver Stiffness measurement (LSM) and serological indicators.
