# Supplementary material for: From Large-Scale Characterization to Subgroup-Specific Predictive Modeling: A Study on the Diagnostic Value of Liver Stiffness Measurements in Focal Liver Lesions
Source: Diagnostics (Basel). 2025 Aug 8;15(16):1986. doi: 10.3390/diagnostics15161986 (PMC12384809; doi:10.3390/diagnostics15161986)
Supplement: Supplementary file 1 [file diagnostics-15-01986-s001.zip › Table S1 and Figure S1.pdf]

Table S1. Spearman's correlation analysis of liver stiffness measurement (LSM) with serological indicators.

Variable	Spearman's rho (ϱ)	*p*-value
Platelet Count (PLT)	-0.390	< 0.001
International Normalized Ratio (INR)	0.370	< 0.001
Aspartate Aminotransferase (AST)	0.363	< 0.001
Prothrombin Time (PT)	0.357	< 0.001
Alpha-fetoprotein (AFP)	0.296	< 0.001
γ-glutamyl transferase (GGT)	0.287	< 0.001
Des-gamma-carboxy prothrombin (DCP)	0.279	< 0.001
Alanine Aminotransferase (ALT)	0.278	< 0.001
Total Bilirubin (TBIL)	0.222	< 0.001

Figure S1. Correlation analysis between liver Stiffness measurement (LSM) and serological indicators.

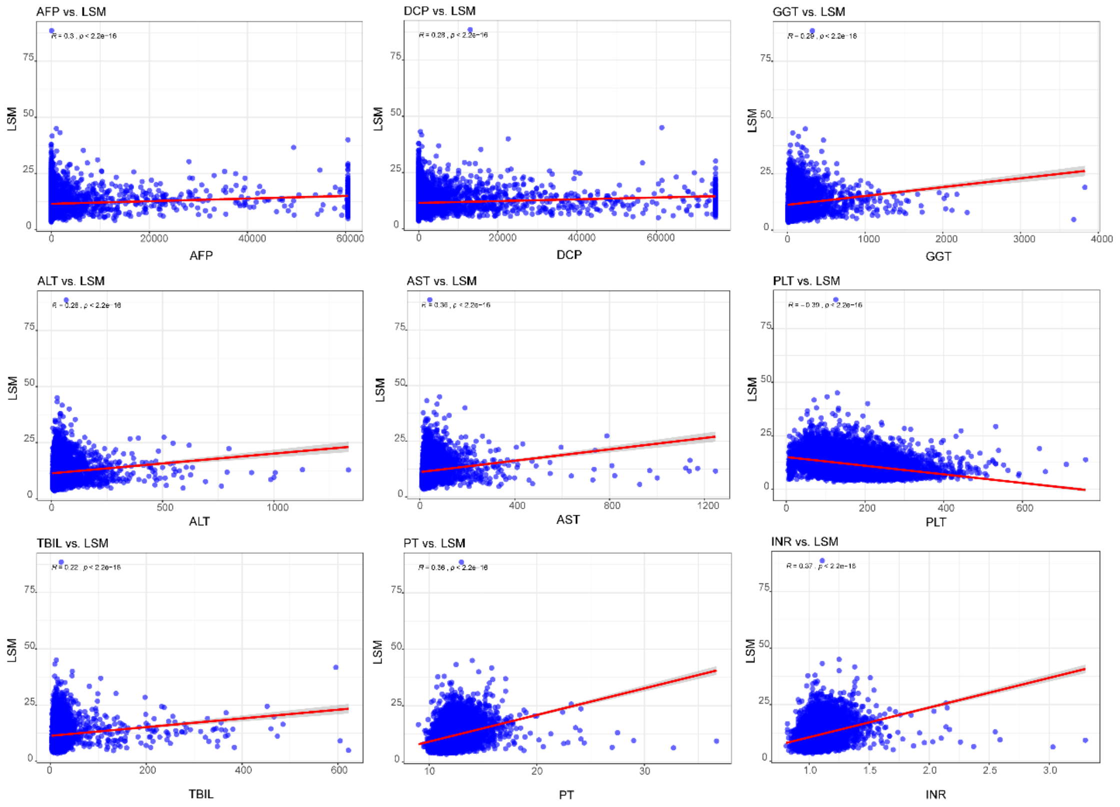

LSM, liver stiffness measurement (kPa); AFP, alpha-fetoprotein (ng/mL); DCP, des-gamma carboxy prothrombin (μg/L); GGT, γ-glutamyl transferase (U/L); ALT, alanine aminotransferase (U/L); AST, aspartate aminotransferase (U/L); PLT, platelet count (×10^9/L); TBIL, total bilirubin (μmol/L); PT, prothrombin time (s); INR, international normalized ratio.
